# Increased Plasma Levels of 8-Hydroxy-2′-deoxyguanosine (8-OHdG) in Patients with Pseudoexfoliation Glaucoma

**DOI:** 10.1155/2019/8319563

**Published:** 2019-07-01

**Authors:** Altaf A. Kondkar, Tahira Sultan, Taif A. Azad, Lubna Tabussum, Essam A. Osman, Saleh A. Al-Obeidan

**Affiliations:** ^1^Department of Ophthalmology, College of Medicine, King Saud University, Riyadh, Saudi Arabia; ^2^Glaucoma Research Chair in Ophthalmology, College of Medicine, King Saud University, Riyadh, Saudi Arabia; ^3^Ambulatory Care, King Saud University, Riyadh, Saudi Arabia

## Abstract

**Purpose:**

To investigate systemic oxidative stress-induced DNA damage in patients with pseudoexfoliation glaucoma (PXG), we estimated plasma levels of 8-hydroxy-2′-deoxyguanosine (8-OHdG) as a marker for oxidative DNA damage in comparison to controls. In addition, we also examined a combined effect of lysyl oxidase-like 1 (*LOXL1*) polymorphism status and 8-OHdG levels on PXG risk.

**Materials and Methods:**

A retrospective case-control study was performed to estimate plasma levels of 8-OHdG in 41 PXG patients and 45 nonglaucomatous controls using the enzyme-linked immunosorbent assay (ELISA). The assay was performed in duplicate on an automated ELISA analyzer. Two common polymorphisms (rs1048661 and rs3835942) in *LOXL1* gene were genotyped by Sanger sequencing.

**Results:**

The mean and median levels of 8-OHdG were significantly increased in the PXG cases (*p*=0.032) and male subjects (*p*=0.041). Subjects with levels greater than the third quartile (75% percentile) exhibited a significant increased risk of PXG (odds ratio = 4.06, 95% confidence interval (CI = 1.11–14.80, *p*=0.029)). Within- and between-group comparisons showed that the mean levels were higher in individuals carrying the *LOXL1* risk variant (G/G), but not statistically significant. In logistic regression analysis, both 8-OHdG (*p*=0.044) and rs3835942 (*p*=0.012) showed a statistically significant effect on the PXG outcome. However, the effect was lost when age, sex, and rs1048661 were included. A significant positive correlation was observed between 8-OHdG levels and intraocular pressure (*R*=0.284, *p*=0.008) and cup/disc ratio (*R*=0.233, *p*=0.031). Furthermore, in receiver operating characteristic analysis, the area under the curve was statistically significant (*p*=0.032) with a value of 0.635 (95% CI = 0.518–0.751).

**Conclusion:**

The study demonstrates an association of increased plasma levels of 8-OHdG in patients with PXG, supporting the role of oxidative stress, and increased oxidative DNA damage in PXG development.

## 1. Introduction

Glaucoma is one of the leading causes of irreversible blindness worldwide [1], including in Saudi Arabia [2]. As a result of blocked aqueous humor outflow at the trabecular meshwork (TM), elevated intraocular pressure (IOP) is considered as a primary risk factor that triggers the death of retinal ganglion cells (RGCs) and progressive loss of RGC axons, causing glaucomatous optic neuropathy [3]. Pseudoexfoliation glaucoma (PXG) is often characterized by accumulation of abnormal fibrillar extraceullular materials in the anterior segment of the eye, primarily along the pupillary border, which is considered as a pathologic hallmark of the disease [4]. In comparison with more common primary open-angle glaucoma (POAG), patients with PXG often show a more aggressive clinical course with higher IOP, larger cupping, severe visual field defects, and worse prognosis with rapid progression of the disease [3, 4]. There is a lack of systematic epidemiological studies in the literature to know the exact prevalence of PXG in Saudi Arabia. However, it has been observed that from approximately 600 new glaucoma cases registered at our glaucoma unit of King Abdulaziz University Hospital in Riyadh, less than 10% of these cases are diagnosed with PXG.

PXG represents a complex and multifactorial adult-onset disease. The disease involves both genetic and environmental factors that contribute to the etiological pathophysiology of the disease [5]. Lysyl oxidase-like 1 (*LOXL1*) is a strong candidate gene reported to contribute to the risk of PXG development in multiple ethnic groups, including Saudi Arabians. *LOXL1* is involved with extracellular matrix formation and stability [5, 6]. There are strong lines of evidence to suggest a role of oxidative stress mechanism(s) in pathogenesis of PXG [7–10]. Oxidative stress is generally induced through the formation of reactive oxygen species (ROS) such as superoxide, peroxide, and hydroxyl radicals that can initiate and propagate free radicals [7]. The accumulation of ROS levels in the cells induces oxidative damage in macromolecules like lipids, proteins, RNA, DNA, and mitochondria, resulting in their cellular dysfunction and/or apoptosis [7]. It has been demonstrated that oxidative stress causes an increase in the IOP by initiating TM degeneration, thereby hindering the aqueous outflow pathway [11]. Mutations, haplogroups, and decreased respiratory activities in the mitochondria have also been associated with various types of glaucoma [12, 13]. Besides, a polymorphism in glutathione S-transferase (*GST*) gene, an enzyme involved in detoxifying peroxidized lipids and various harmful toxins, has also been associated with glaucoma [14]. Reduced GST activity may interfere with detoxification of oxidative metabolites and aggravate the damaging effects of oxidative stress on the optic nerve [14]. The total antioxidant status (TAS) of biological samples is an important indicator of oxidative stress and a useful tool to predict oxidative status [9]. Our previous study has shown that plasma TAS levels were significantly low in PXG patients as compared to controls, supporting the role of oxidative stress in the pathogenesis of PXG [9]. Besides, we also reported a combined effect of the *LOXL1* alleles and the decreased TAS that may contribute to the overall risk of PXG. In humans, in vivo experiments have demonstrated that oxidative DNA damage is significantly more abundant in the TM cells of glaucoma patients. In addition, both increased IOP and visual field damage were significantly related to the amount of oxidative DNA damage affecting TM cells [11, 15]. Oxidative stress/ROS can induce breaks or base modifications in the DNA resulting in the release of DNA oxidation products, including 8-hydroxy-2′-deoxyguanosine (8-OHdG) [16]. 8-OHdG is one of the multiple products of DNA oxidation that can be easily quantified and is commonly used as a biomarker to assess oxidative DNA damage [17].

The aim of this study was to investigate systemic oxidative stress-induced DNA damage in patients with PXG. We estimated plasma levels of 8-OHdG as a marker for oxidative DNA damage and compared it with nonglaucomatous healthy controls. In addition, we also investigated the combined effect of *LOXL1* polymorphism status and 8-OHdG level on the risk of PXG.

## 2. Materials and Methods

### 2.1. Study Population

The study adhered to the tenets of the Declaration of Helsinki and was approved by the institutional review board and research ethics committee (approval number # 08–657). Following written informed consent, participants of Saudi origin with established clinical diagnosis of PXG (*n*=41) and ethnically matched healthy controls (*n*=45) were recruited for the study at King Abdulaziz University Hospital in Riyadh, Saudi Arabia. The inclusion-exclusion criteria of patients and controls have been described previously [10].

### 2.2. Plasma and DNA Preparation

Plasma samples were obtained from EDTA blood following centrifugation at 5500 ×g for 5 min. DNA was extracted from the buffy layer using the illustra blood genomicPrep Mini Spin Kit (GE Healthcare, Buckinghamshire, UK). Storage was at −80°C until use.

### 2.3. Estimation of Plasma Levels of 8-Hydroxy 2′-Deoxyguanosine

The estimation of 8-OHdG levels was performed using a commercial kit (Trevigen, Gaithersburg, MD, USA) based on a competitive sandwich enzyme-linked immunosorbent assay (ELISA). The assay was performed in duplicate on a ChemWell-T automated ELISA analyzer (Awareness Technology Inc., FL, USA), as per the manufacturer's instructions. The 8-OHdG levels were established utilizing the standard curve and expressed in ng/mL.

### 2.4. *LOXL1* Sanger Sequencing

DNA samples were sequenced for the two common *LOXL1* gene polymorphisms (rs1048661 and rs3825942) using primers and amplification conditions as described elsewhere [9].

### 2.5. Statistical Analysis

Data are presented as mean ± SD and median for continuous variables and as counts and percentages for categorical variables. Normality testing for 8-OHdG levels was done using the Kolmogorov–Smirnov test. Mean differences between groups were tested by Student's *t*-test. The Mann–Whitney *U* test was used to compare median values between the patients and controls. The categorical variables were tested by the chi-square test and Fisher's exact test where applicable. The correlation testing was done using Pearson's method. A binary logistic regression analysis was performed to estimate the impact and effect of mean 8-OHdG levels and other risk factors on disease outcome. A receiver operating characteristic (ROC) curve was generated, and the area under the curve (AUC) value was analyzed by the Mann–Whitney test. Odds ratio (OR) was calculated, and a confidence interval (CI) was set to 95%. All statistical tests were two-sided, and a *p* value less than 0.05 was considered statistically significant. Statistical analysis was performed with SPSS version 19.0 (IBM Corp., Armonk, New York, USA) and StatView software version 5.0 (SAS Institute, Cary, NC).

## 3. Results

### 3.1. Study Population and 8-OHdG Levels

**Figure 1:**
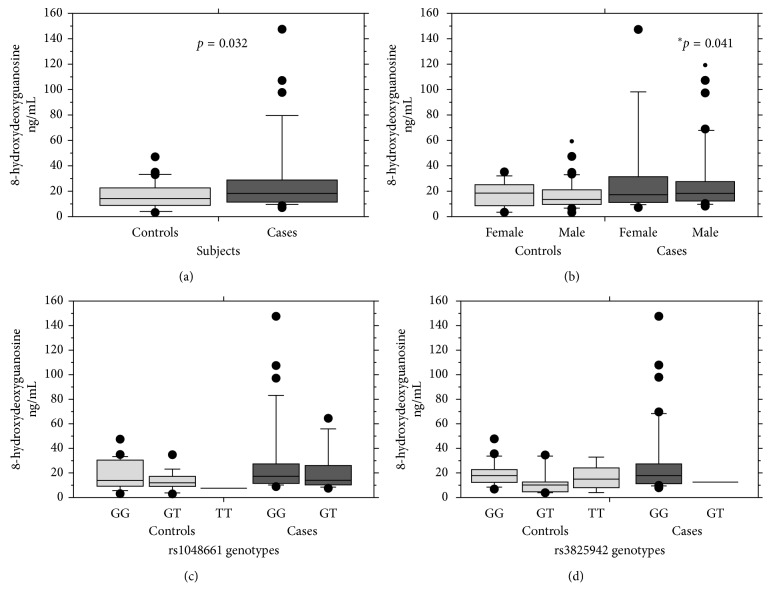
Box plot showing distribution of 8-hydroxydeoxyguanosine levels between cases and controls of (a) pseudoexfoliation glaucoma (PXG), (b) males and females, (c) *LOXL1* rs1048661 genotypes, and (d) *LOXL1* rs3825942 genotypes. Differences were tested by the Mann–Whitney *U* test both within and between the study groups. Significance was observed only between cases and controls and between control males vs. PXG males as indicated in (a) and (b), respectively. No other significant distribution was observed.

As shown in Table 1, there was no significant difference between PXG cases and controls for age, gender, systemic disease status, smoking, and family history of glaucoma. Normality testing for 8-OHdG levels showed a skewed distribution (*p* < 0.001). Both mean and median 8-OHdG levels were significantly elevated in the PXG cases and male subjects as compared to the controls (Table 1). Figures 1(a) and 1(b) show the box-plot representation of 8-OHdG levels according to disease status and gender distribution, respectively.

### 3.2. Levels of 8-OHdG and Risk of PXG

To assess the risk of PXG associated with increasing levels of 8-OHdG, the 8-OHdG concentrations were dichotomized (uncategorized as cases and controls) at the 50^th^ percentile (or median value) and by quartiles (Table 2). The overall median cutoff of the 8-OHdG level was 17.68 ng/mL. Although cases showed an increased risk of disease at this level (OR = 1.76, 95% CI = 0.75–4.15), the difference was nonsignificant (*p*=0.190). Similarly, using quartile distribution, two cutoff levels identified were 10.08 ng/mL (the first quartile or the 25^th^ percentile) and 27.72 ng/mL (the third quartile or the 75^th^ percentile). Using these two cutoff values, subjects were then categorized into three groups: less than the first quartile, the interquartile, and greater than the third quartile (Table 2). Overall, there was no significant additive effect of increasing levels of 8-OHdG and PXG outcome (*χ*^2^ = 4.87, df = 2; *p*=0.0875). In addition, compared to 8-OHdG levels less than the first quartile (<10.08 ng/mL), subjects with interquartile levels showed a nonsignificant increased risk of disease (OR = 2.50, 95% CI = 0.81–7.63, *p*=0.103), whereas subjects with levels greater than the third quartile (75^th^ percentile) exhibited a significant increased risk of PXG (OR = 4.06, 95% CI = 1.11–14.80, *p*=0.029).

### 3.3. 8-OHdG Levels and *LOXL1* Polymorphisms

We further investigated the genotype effect of polymorphisms rs1048661 (g.5758 G>T) and rs3825942 (g.5758 G>A) in the *LOXL1* gene on 8-OHdG levels in PXG cases and controls. Overall, as shown in Table 3, there was no significant difference between 8-OHdG levels and different genotypes for both *LOXL1* polymorphisms (Figures 1(c) and 1(d)).

For rs1048661, G/G was the most common genotype, followed by G/T and T/T. No T/T homozygosity was observed in the patient group as compared to only one in the controls. The mean 8-OHdG levels were observed to be highest in the G/G genotypes as compared to G/T, T/T, or G/T + T/T groups. However, these levels did not vary significantly both within and between the study group comparisons.

Similarly, for rs383592, G/G was the most prevalent genotype, followed by G/A and A/A genotypes, with the latter being absent in PXG patients. G/G was the most common genotype exhibiting highest levels of 8-OHdG in comparison to G/A, A/A, or G/A + A/A groups. The levels did not vary significantly between cases and controls for G/G genotypes (*p*=0.369). In addition, a within-group comparison in controls also did not show any significant genotype effect on 8-OHdG for G/A (*p*=0.061), A/A, (*p*=0.818) and G/A + A/A (*p*=0.118) as compared to G/G genotype.

### 3.4. 8-OHdG Levels and Other Risk Factors

With a view to examine the effect of age, sex, *LOXL1* polymorphisms, and 8-OHdG levels in patients with PXG, a binary logistic regression analysis was performed using diseased/nondiseased as a dependent variable (outcome). The analysis showed that both 8-OHdG (*p*=0.044) and rs3835942 (*p*=0.012) have statistically significant effect on the disease outcome. However, in a combined analysis with age, sex, 8-OHdG, rs1048661, and rs3835942, none of these risk factors showed any significant impact on PXG (Table 4).

### 3.5. Correlation between 8-OHdG and Other Glaucoma Indices in PXG Patients

A significant positive correlation was observed between 8-OHdG and IOP (*R*=0.284, *p*=0.008) and cup/disc ratio (*R*=0.233, *p*=0.031), as opposed to none with age (*R*=0.154, *p*=0.157) and number of antiglaucoma medication (*R*=−0.035, *p*=0.829).

### 3.6. ROC Curve and 8-OHdG

ROC curve analysis of 8-OHdG levels in PXG patients and controls revealed an AUC of 0.635 (95% CI = 0.518–0.751) that was statistically significant (*p*=0.032), indicating that the plasma levels of 8-OHdG could satisfactorily discriminate between PXG patients and controls.

## 4. Discussion

Increased ROS, oxidative damage, and imbalance between prooxidant status and antioxidant status are critical factors which significantly contribute to glaucomatous neurodegeneration [7, 18]. This study reports increased levels of systemic 8-OHdG, a marker of oxidative stress-induced DNA damage, in patients with PXG.

DNA damage can be inflicted by both extrinsic and intrinsic agents such as ionizing radiations, ultraviolet light, toxic chemicals/metal ions, and ROS generated as a consequence of normal cellular metabolism of oxygen [19]. The endogenous processes that may plausibly contribute to an ongoing DNA damage *in vivo* include oxidative methylation, depurination, and deamination [16, 19]. ROS-induced damages include base and sugar lesions, protein, and DNA cross-links, and single-strand/double-strand breaks. Moreover, the base guanine is most susceptible to oxidative modifications because of its least redox potential [16, 19]. The most common byproduct formed due to oxidative modification of guanine by hydroxyl radical is 8-hydroxyguanine (8-OH-Gua) and its 2′-deoxynucleoside equivalent, 8-OHdG, which is proposed to be an excellent marker for oxidative damage to DNA [20]. A number of studies have provided strong evidence for association between increased levels of 8-OHdG and glaucomatous optic neuropathy [11, 15, 21].

Initial studies on human TM specimens obtained during filtration surgery have demonstrated presence of high levels of 8-OHdG in patients with glaucoma [11, 15]. In a study by Sorkhabi et al. that included 15 POAG and 13 PXG patients, both aqueous humor (AH) and serum 8-OHdG levels were high in patients as compared to controls [21]. Similarly, high levels of serum 8-OHdG have been reported in patients with primary angle closure glaucoma as compared to normal subjects [22]. Yuki and Tsubota reported an increased urinary 8-OHdG/creatinine level to be associated with glaucomatous visual field progression in subjects with normal tension glaucoma [23]. Likewise, in another recent study by Mohanty et al., both plasma and AH 8-OHdG levels were significantly higher in POAG patients in comparison to cataract controls [24]. This effect was attributed to reduced expression of DNA repair enzymes of the base excision repair pathway [24]. In addition, the study also reported a strong positive correlation between systemic (plasma) 8-OHdG levels and AH 8-OHdG levels, suggesting that systemic 8-OHdG levels could be predictive of local 8-OHdG levels in the eye [24].

The exact role of 8-OHdG and/or oxidative stress leading to the development and progression of glaucomatous optic neuropathy is still speculative. 8-OHdG is among the best characterized oxidative lesions, and it can give rise to C : G to A : T transversion mutations [25]. Some lesions in DNA are subjected to cellular repair by *in vivo* DNA repair mechanisms that cleave off the damaged DNA [25]. However, failure to repair these damages can have serious biological implications and may lead to carcinogenesis or development of neurodegenerative disorders [20, 24, 26, 27]. 8-OHdG can also significantly induce telomere shortening that may contribute to physiological and pathological conditions *in vivo* [25, 28]. Furthermore, it has been suggested that 8-OHdG may have an epigenetic-like regulatory role in cells undergoing oxidative stress in regulation of gene transcription [25]. Moreover, the abnormal effects of oxidative stress and ROS in glaucoma pathogenesis via increased IOP and/or hypoxia, TM degeneration, glial cell damage, autophagy, mtDNA damage, nuclear-kappa B activation, peroxynitrite stress, and ocular hemodynamics stimulating apoptosis and inflammatory pathways to promote RGC death and optic nerve damage have been well documented [7]. Increased levels of 8-OHdG observed in our study may plausibly contribute to PXG pathogenesis by similar mechanism(s).

A strong association of two missense variants, rs1048661 and rs3825942, in the *LOXL1* gene with PXG has been consistently replicated in multiple ethnic groups including Saudi Arabians [5, 6]. A complete understanding of this genetic association to PXG pathology is still not known; however, a number of mechanisms have been postulated. These include its effect on elastin formation, altered *LOXL1* expression, splicing-effect, or possibility of their association/linkage with an actual unidentified functional allele [5]. We investigated the genotype effects of these two common variants on 8-OHdG and their combined effect on the risk of PXG. The genotypes were not found to influence the 8-OHdG levels, although the levels were high in the risk (G/G) variants. Both 8-OHdG and rs3835942 were found to be significant risk factors for PXG. However, this significance was lost in a combined analysis that also included age, sex, and rs1048861 genotypes. A lack of sufficient numbers in each genotype group could be a likely explanation to this loss and absence of any significant effect.

An increased IOP and visual field damage have been significantly correlated to the amount of oxidative DNA damage in the TM cells [11, 15]. Similarly, the IOP and cup/disc ratio also showed a significant positive correlation with 8-OHdG levels in our study, suggestive of its possible utility as a marker of disease severity. Besides, a number of reports have supported an existence of inverse relation between antioxidant defense mechanism(s) and oxidative stress in the pathophysiology of glaucoma [9, 18, 21, 29]. Our group has also previously reported significantly reduced plasma total antioxidant status (TAS) in PXG patients as compared to controls [9]. Interestingly, TAS levels were available for the PXG patients (not for controls; data not shown) included in this study that showed a significant negative correlation between 8-OHdG and TAS in PXG patients (*R*=−0.365, *p*=0.022), supporting the plausible role of increased oxidative stress and decreased antioxidant defense mechanisms in PXG.

The findings of this study require cautious interpretation because of its certain limitations. First, we have to acknowledge the fact that the systemic increase in 8-OHdG may not exactly reflect the situation in the anterior chamber of the eye, where cells/tissues are constantly exposed to a higher amount of free radical insults and thus are more directly involved in the development and progression of glaucoma through the oxidative stress mechanisms. This would require an additional validation in AH samples. Second, the study is purely descriptive in nature and does not provide any temporal or mechanistic evidence or suggest any causal implications of elevated 8-OHdG in PXG. Finally, a relatively small number of samples have been examined in this study. A replication in a much larger cohort would certainly strengthen the observations of this study. Nonetheless, considering a mean difference (effect size) of 13 ng/mL in 8-OHdG concentration as observed between PXG cases and controls, with an average standard deviation of 20 and type I error of 0.05 (two-sided), the study exhibits a power of >80%.

## 5. Conclusion

To conclude, the study demonstrates an increased level of systemic 8-OHdG in patients with PXG, supporting an association of this marker with PXG, and the plausible role of oxidative stress and increased oxidative DNA damage in PXG etiology.

## Figures and Tables

**Table 1:** Demographic characteristics and 8-hydroxydeoxyguanosine (8-OHdG) levels between pseudoexfoliation glaucoma cases and controls.

Variables	Cases (*n*=41)	Controls (*n*=45)	*p*value
Age in years, mean (SD)	62.27 (6.31)	59.91 (8.33)	0.146^a^
Male/female, *n*	26/15	30/15	0.751^b^
Systemic diseases, *n* (%)			
Diabetes mellitus	6 (14.6)	6 (13.3)	0.862^b^
Hypertension	4 (9.7)	5 (11.1)	0.605^b^
Coronary artery disease	2 (4.8)	2 (4.4)	0.924^b^
Hypercholesterolemia	3 (7.3)	2 (4.4)	0.569^b^
Family history of glaucoma	5 (12.2)	1 (2.2)	0.069^b^
Smokers	5 (12.2)	5 (11.1)	0.874^b^

*8-OHdG concentration, ng/mL*			
Mean (SD)	30.48 (31.52)	16.95 (10.66)	0.008^a^
Median	18.11	13.78	0.032^c^

*By gender*			
Males, mean (SD)	29.53 (26.94)	17.02 (11.02)	0.023^a^
Median	18.17	13.12	0.041^c^
Females, mean (SD)	32.12 (39.22)	16.80 (10.29)	0.154^a^
Median	17.68	17.97	0.442^c^

*Notes*. ^a^Independent sample *t*-test (two-tailed); ^b^chi-square test; ^c^Mann–Whitney *U* test.

**Table 2:** Evaluation of risk of pseudoexfoliation glaucoma according to median and quartile levels of 8-hydroxydeoxyguanosine (8-OHdG).

8-OHdG ng/mL	No. of cases (%)	No. of controls (%)	Odds ratio	95% confidence interval	*p* value^a^
*By median*					
<17.68	17 (41.4)	25 (55.5)	Reference	—	—
≥17.68	24 (58.5)	20 (44.5)	1.76	0.75–4.15	0.190

*By quartiles * ^†^					
<10.08	6 (14.6)	15 (33.3)	Reference	—	—
10.08–27.72	22 (53.6)	22 (48.8)	2.50	0.81–7.63	0.103
>27.72	13 (31.7)	8 (17.8)	4.06	1.11–14.80	0.029

*Note.* ^a^Chi-square test; ^†^first quartile (<25^th^ percentile); interquartile (25^th^–75^th^ percentile); third quartile (>75^th^ percentile).

**Table 3:** Levels of 8-hydroxydeoxyguanosine (8-OHdG) according to *LOXL1* rs1048661 and rs3835942 polymorphisms in pseudoexfoliation glaucoma cases and controls.

*LOXL1* genotypes	8-OHdG ng/mL, mean (SD)		*p* value
	Controls^*∗*^	Cases^*∗*^	

*rs1048661*			
G/G	18.55 (12.04)	30.16 (17.90)	0.236
G/T	13.71 (8.16)	21.99 (19.31)	0.617
T/T	7.84	—	—
G/T + T/T	13.26 (7.98)	21.99 (19.31)	0.469

*rs3825942*			
G/G	18.53 (10.68)	28.89 (30.97)	0.369
G/A	12.93 (11.46)	11.83	—
A/A	16.13 (11.33)	—	—
G/A + A/A	14.00 (11.11)	11.83	—

*Note*. ^*∗*^*p* values tested within groups using G/G as reference by the Mann–Whitney *U* test were also nonsignificant (*p* > 0.05).

**Table 4:** Binary logistic regression analysis of risk variables on disease outcome.

Risk variables	Odds ratio (95% confidence interval)	*p* value
Age	1.035 (0.964–1.112)	0.345
Sex^a^	1.150 (0.352–3.762)	0.817
8-OHdG^b^	1.035 (0.985–1.053)	0.292
rs1048661^c^	—	0.599
rs3835942^c^	—	0.073

*Note.* ^a^Females as reference; ^b^ 8-OHdG, 8-hydroxydeoxyguanosine; ^c^ G/G genotypes as reference.

## Data Availability

The data supporting the conclusions of this article are all presented within the article.
